# The Efficacy of Hydrofit and Spongel in the Management of Venous Injury

**DOI:** 10.3400/avd.nmt.25-00073

**Published:** 2025-08-30

**Authors:** Takayuki Kawashima, Takashi Shuto, Kazuki Mori, Hidetaka Yamauchi, Takeshi Wada, Shinji Miyamoto

**Affiliations:** Department of Cardiovascular Surgery, Oita University, Yufu, Oita, Japan

**Keywords:** Hydrofit, AQUABRID, venous injury

## Abstract

Intraoperative venous bleeding, particularly from deep pelvic veins, can be difficult to control with suturing or standard compression. We introduce the “French toast method,” a hemostatic technique that combines Hydrofit (Terumo, Tokyo, Japan) with a gelatin sponge (Spongel; LTL Pharma, Tokyo, Japan). A small amount of Hydrofit is spread onto the sponge, which is then applied to the bleeding site. Immediately afterward, saline is poured over the area to activate Hydrofit, followed by fingertip compression. This method enables rapid and secure hemostasis without the need to remove a silicone sheet, thereby reducing the risk of rebleeding and simplifying management of difficult venous hemorrhage.

## Introduction

Intraoperative venous injuries—especially those involving deep pelvic veins such as the iliac vein—can lead to significant hemorrhage. Achieving hemostasis through suturing is often challenging, and conventional compression techniques may require prolonged pressure while still posing a risk of rebleeding.

Hydrofit (Terumo, Tokyo, Japan) is a nonabsorbable topical hemostatic agent developed in Japan. It is composed of a synthetic, non-biological polyurethane material that reacts with moisture in the blood to form a film over the bleeding site, thereby providing hemostasis independent of the patient’s coagulation ability.^1)^ Owing to its hemostatic properties, Hydrofit has been commercially available in Japan since 2014 for use in aortic surgery and has been marketed internationally as AQUABRID since 2019. We have previously reported the effectiveness of a hemostatic technique that we refer to as the “French toast method,” which combines Hydrofit with a gelatin sponge, Spongel (LTL Pharma, Tokyo, Japan).^2)^ We have applied this method to achieve hemostasis in cases of bleeding associated with venous injuries and have obtained favorable results. In this report, we describe the technique and present our outcomes.

## New Methods

When encountering bleeding due to venous injury during surgical procedures, initial hemostasis is typically achieved through digital compression. If adequate hemostasis can be obtained using a single finger, the French toast method—which combines Hydrofit and Spongel—can be effectively applied.

**Fig. 1:**
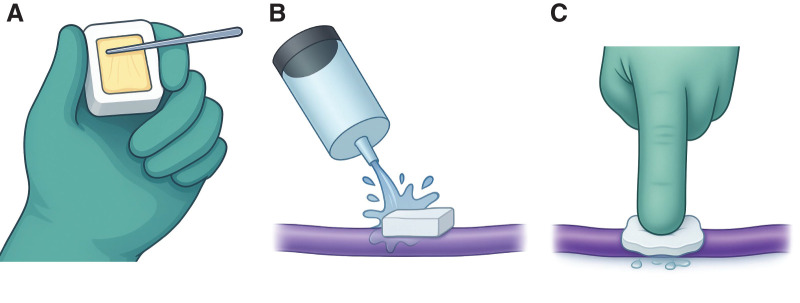
Illustration of the French toast method for hemostasis. (**A**) A small amount of Hydrofit (Terumo, Tokyo, Japan) is thinly spread onto a suitably sized piece of Spongel (LTL Pharma, Tokyo, Japan) using the provided stainless spatula. (**B**) The Hydrofit-coated Spongel is applied to the bleeding site, and a sufficient amount of saline is poured over it. (**C**) The softened Spongel is pressed against the bleeding point with a fingertip. Hemostasis is typically achieved after approximately 60 seconds of compression.

First, the Spongel is trimmed to an appropriate size to match the bleeding point—typically 2.5 × 2.5 cm. A small amount of Hydrofit (approximately 0.4 mL) is then thinly spread over one surface of the Spongel using the provided stainless steel applicator, in a manner similar to spreading butter on bread (**Fig. 1A**). The coated surface of the Spongel is gently applied to the bleeding site, and a sufficient amount of saline (approximately 30 mL) is immediately poured over it (**Fig. 1B**). Once the sponge softens to the consistency of an earlobe, firm compression is applied directly over the bleeding site using a fingertip (**Fig. 1C**). After approximately 60 seconds, the surrounding fluid and any excess Spongel are aspirated. This leaves a thin layer of gelatin sponge impregnated with Hydrofit at the bleeding site, thereby achieving hemostasis.

**Fig. 2:**
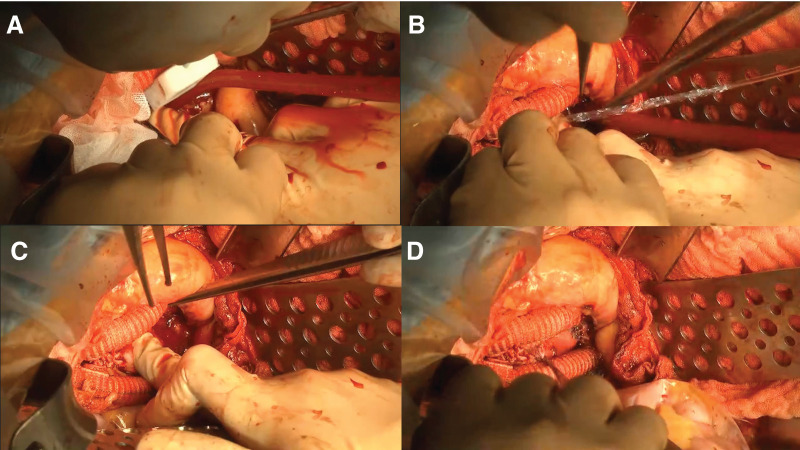
Intraoperative image of the French toast method. (**A** and **B**) The Spongel (LTL Pharma, Tokyo, Japan), thinly coated with Hydrofit (Terumo, Tokyo, Japan), was pressed onto the bleeding site while simultaneously irrigating the area with a generous amount of saline. (**C** and **D**) Hemostasis was achieved after approximately 40 seconds of compression.

A 66-year-old man with a rapidly expanding abdominal aortic aneurysm measuring up to 54 mm in maximum short-axis diameter underwent elective open abdominal aortic repair. Through a midline laparotomy, aortic cross-clamping was performed below the renal arteries. The graft was anastomosed to the right common iliac artery and to both the left external and internal iliac arteries. The anastomoses were completed uneventfully. However, during hemostatic maneuvers, the right iliac vein was accidentally injured with electrocautery. Primary repair with 5-0 polypropylene sutures was attempted, but the friable venous wall tore further, exacerbating the bleeding, and the attempt was abandoned. Manual compression with Surgicel (Ethicon, Somerville, NJ, USA) using a single finger for approximately 3 min did not reduce the bleeding. Therefore, we elected to use the French toast method. While maintaining compression, a Spongel was trimmed to approximately 2.5 × 2.5 cm, a thin layer of Hydrofit was applied to its surface, and the sponge was placed over the bleeding site (**Fig. 2A**). A generous amount of saline was poured simultaneously (**Fig. 2B**), and digital compression was applied (**Fig. 2C**). After 40 seconds, compression was released, and no rebleeding was observed. Removal of surrounding fluid and excess sponge revealed secure and complete hemostasis (**Fig. 2D**). This procedure is demonstrated in detail in the **Supplementary Video**.

## Discussion

Hydrofit has been used in Japan since 2014 as a novel hemostatic agent that functions independently of the patient’s coagulation ability. Its primary characteristic is its ability to react with moisture to achieve hemostasis,^1)^ and it is commonly used for bleeding from needle holes at aortic anastomoses or for oozing-type hemorrhages. However, when using the provided silicone sheet for hemostasis, we have experienced cases in which the Hydrofit layer is unintentionally peeled off along with the sheet, resulting in rebleeding. This can make its use cumbersome and unreliable in certain situations.

To address this issue, we developed a technique referred to as the French toast method.^2)^ In this approach, a gelatin sponge (Spongel) is used in place of the silicone sheet. As a result, there is no need to remove the sponge after achieving hemostasis, thereby eliminating the risk of dislodging the Hydrofit and inducing rebleeding. Additionally, unlike the impermeable silicone sheet, the gelatin sponge allows fluid to pass through, enabling the application of saline to accelerate the chemical reaction of Hydrofit with moisture.

In a simulated vascular circuit experiment, we confirmed that reliable hemostasis could be achieved within 60 seconds.^2)^ Compared to the traditional method using a silicone sheet, this technique offers faster hemostasis and reduces the risk of rebleeding—its greatest advantage. Initially, we applied this technique to aortic bleeding; however, we have since extended its use to venous injuries. During abdominal aortic aneurysm repair, the iliac arteries are often adherent to surrounding structures, and inadvertent injury to nearby veins may result in significant bleeding. In certain cases, bleeding arises from deep pelvic veins, making suture hemostasis technically difficult and time-consuming.

We believe that this technique is particularly effective in such challenging situations. Since January 2020, we have applied this method in over 20 cases of venous injury, including injuries to the iliac, renal, inferior vena cava, hepatic, and splenic veins. In most cases, hemostasis was successfully achieved using this method alone. In cases involving larger venous defects, partial suturing to reduce the size of the defect allowed the French toast method to be applied effectively. In addition, no postoperative rebleeding or other adverse events attributable to this technique have been observed. Similar reports have described successful hemostasis in iliac vein,^3)^ coronary sinus,^4)^ and left ventricular bleeding^5)^ without the use of the provided silicone sheet, instead utilizing the patient’s own arterial wall or adjunctive materials such as Surgicel (Ethicon). These findings suggest that the principles of the French toast method may be broadly applicable to various types of bleeding beyond venous injuries.

Unfortunately, gelatin sponges are no longer available in Japan. As a result, we have been performing the French toast method using Surgicel folded into quarters to match the size of Spongel. We have observed hemostatic efficacy comparable to that achieved with Spongel, and we believe that this method remains a reliable and effective hemostatic technique.

In conclusion, we believe this technique is highly useful for achieving hemostasis in venous bleeding where suture repair is difficult. It should be considered as a bailout strategy in cases of refractory venous hemorrhage.

## Supplementary Infomation

Supplementary Video Intraoperative demonstration of the French toast method using Hydrofit and Spongel for venous hemostasis.
